# Toxic Effects of Methotrexate on Rat Kidney Recovered by Crocin as a Consequence of Antioxidant Activity and Lipid Peroxidation Prevention

**DOI:** 10.29252/ibj.24.1.39

**Published:** 2019-08-28

**Authors:** Cyrus Jalili, Ali Ghanbari, Shiva Roshankhah, Mohammad Reza Salahshoor

**Affiliations:** 1 Medical Biology Research Center, Department of Anatomical Sciences, Kermanshah University of Medical Sciences, Daneshgah Ave., Taghbostan, Kermanshah, Iran;; 2 Department of Anatomical Sciences, Medical School, Kermanshah University of Medical Sciences, Kermanshah, Iran

**Keywords:** Antioxidants, Crocin, Kidney, Methotrexate

## Abstract

**Background:**

The application of MTX as a chemotherapy agent and immune system suppressant has various side effects. Crocin, a xanthine derivative plant, has many therapeutic benefits. This study was planned to assess the effect of crocin on renal toxicity of MTX in a rat model.

**Methods:**

Forty eight rats were divided randomly into eight groups (n = 6), which received normal saline, MTX, crocin, and MTX + crocin for 28 days intraperitoneally. The levels of oxidative stress in kidney and blood serum were measured, and the kidney was analyzed histologically.

**Results:**

MTX caused an enhancement in the levels of thiobarbituric acid reactive substances and biochemical marker (creatinine and BUN). Besides, a significant decrease was observed in tissue parameters and antioxidant capacity compared to the normal control group (*p* < 0.001). The crocin and crocin + MTX decreased the biochemical markers, the levels of thiobarbituric acid reactive species, and tissue parameters considerably at entire dose (12.5, 25, and 50 mg/kg) and enhanced the antioxidant capacity levels compared to the MTX group (*p* < 0.001).

**Conclusion:**

Administration of crocin improves the damage caused by MTX in rats. The crocin by the establishment of balance in the levels of antioxidant prevents the damage to the renal cell membrane, and subsequently the renal damage repairs.

## INTRODUCTION

Although administration of chemical drugs are effective in the treatment of many diseases, long-term and even cross-sectional use can have many side effects.^[^^1^^]^. Hence, in recent years, the application of medicinal plants as an antioxidant, among which saffron with a special and great place in the food industries, has increasingly been considered^[^^2^^]^. 

Saffron is a small, perennial bulbous plant belonging to the Lily family with the height of 10-30 cm^[^^3^^]^. It is the native plant of Iran and the Mediterranean countries^[^^4^^]^. In addition to the application as a popular food additive, saffron is used in traditional medicine for laxative, and also as an appetizer, a sedative, a muscle relaxant, and a therapeutic agent in hepatic disorders, asthma, bronchitis, nausea, and cardiovascular disorders. The role of saffron in the elimination of dysentery and infection in urinary tract is proved^[^^5^^]^. 

Crocin, the compound of the bitter taste of saffron, is a glycoside containing a carotenoid called crocetin, which determines the color of saffron^[^^6^^]^. The results of Erdemli *et al.*'s^[^^7^^]^ study have indicated that crocin can prevent oxidative damage in renal ischemic-injuries of rats owing to its antioxidant effects. Crocin can also protect the liver against oxidative stress-induced damage^[^^8^^]^. Antioxidants are substances that can protect the body against different types of oxidative stresses, even in small amounts^[^^9^^]^. Renal glomeruli seem to be highly sensitive to the oxidative stress^[^^10^^]^; thus, antioxidant administration with the effect of free radical scavenging can reduce the severity of obstructive nephropathies^ [^^11^^]^. 

MTX is one of the most commonly used drugs frequently recommended to treat some types of rheumatic diseases^[^^12^^]^. MTX is also used as a folate antagonist in high doses to treat certain cancers^[^^13^^]^. Reports have displayed that the reduction in the concentration of cellular antioxidant enzymes results in the sensitivity of the affected cells to oxidative damage^[^^14^^]^. Although the MTX mechanisms of action are still not well known, it is assumed that this drug plays a main role in decreasing the effectiveness of the antioxidant defense system and also in increasing the sensitivity of cells to ROS by reducing glutathione levels^[^^15^^]^. MTX can suppress DNA replication in normal and malignant cells^[^^16^^]^ and can increase the expression of p53 transcription factor^[^^17^^]^. According to the results of a study conducted by Abraham *et al.*^[^^18^^]^, MTX can lead to the oxidative stress and renal damage. The aim of this study was to determine the anti-degenerative effects of crocin on kidney against MTX renal toxicity in male rats.

## MATERIALS AND METHODS

**Animals**

In this experimental study, 48 male Wistar rats (weighing 220-250 g) were purchased from Pasteur Institute of Iran (Tehran) and transferred to the animal house in Kermanshah University of Medical School, Kermanshah, Iran. During the study, the animals were kept in standard conditions in special cages and on a straw bed at 22 ± 2 °C for 12-hour light/12-hour dark. Municipal water and standard food were freely given to the animals, and the experiments started after one-week adaptation. The present investigation was in compliance with ethical and human principles of research and approved by the Ethics Committee of Kermanshah University of Medical Sciences (ethical code no. 1396.435).

**Study groups and treatment of animals**

Male rats (n = 48) were randomly classified into eight groups of six each. The first group (normal control group) received normal saline equivalent to the amount of other injections. The second group (control) received 20 mg/kg of MTX (dissolved in normal saline). In the third to fifth groups, the crocin was administered at the doses of 12.5, 25, and 50 mg/kg, respectively. All rats in groups six to eight received MTX (20 mg/kg) along with crocin at the doses of 12.5, 25, and 50 mg/kg, respectively. All the injections were carried out intraperitoneally once a day for 28 days^[^^8^^,^^19^^]^.

**Dissection and sampling**

 A day after the last injection, the rats were anesthetized with a mixture of ketamine HCl (100 mg/kg) and xylazine (10 mg/kg), and the blood serum and kidney samples were collected. The blood samples were incubated at 37° C for 15 minutes to form a clot. The clotted blood was then centrifuged at 1500 ×g for 15 minutes to collect the serum samples. The separated sera were kept at -70° C until biochemical evaluations. The removed kidneys were maintained in 10% buffered formaldehyde for more histological and morphometric analyses^[^^20^^]^.

**MDA measurement**

The thiobarbituric acid reactive species were measured by MDA using colorimetric analysis to assess the oxidative stress as the last product of lipid peroxidation in the renal tissue. Briefly, 1400 μl of each of acetic acid, thiobarbituric acid, and sodium dodecyl sulfate (all from Sigma, USA) was added to 100 μl of the homogenated kidney samples, and the mixture was incubated at 37 °C for 50 min. Subsequently, 4 ml of 1-Butanol (Sigma) was added to the mixture and vortexed for 2 min at 3000 g for 15 min. The absorbance of the upper layer was measured at 532 nm (Spectro, Germany), and consecutive concentrations of 1,1,3,3-tetraethoxypropane (5, 10, 25, 50, 75, and 100 μM; Sigma) were used as the external standard^[^^21^^]^.

**FRAP**

FRAP test was used to measure the antioxidant capacity of the kidney. The FRAP substance was prepared by mixing 30 ml of acetate buffer and 1.5 ml of ferric chloride (both from Sigma). A volume of 60 μl of kidney homogenate was added to 1.5 ml of fresh prepared solution. The reaction was incubated at 37 °C for 10 min, and the absorbance of the blue-colored complex was measured against a blank at 593 nm. Serial concentrations of FeSO_4_.7H_2_O (Sigma) were used as the external standard^[^^21^^]^.

**Assessment of renal index**

The body weight of the animals was first measured at the end of the study to calculate the renal index. Following the removal of kidneys, the mean weights of the kidneys were calculated, and the kidney index was calculated by dividing the total left and right kidney weights to the total body weight of the rats^[^^11^^]^.

**Biochemical assay**

The serum samples were analyzed for creatinine and BUN using an autoAnalyzer (RA 1000; Technicon Instruments, USA)^[^^20^^]^. 

**Tissues preparation and quantitative measurements of renal glomeruli**

The renal samples were fixed by 10% buffered formaldehyde solution and washed with distilled water. The kidneys were divided into two equal parts by a cross-sectional cut from the middle line. The tissues were processed according to the routine histology protocol, including dehydration by ascending concentrations of alcohol, clearing, and infiltration with paraffin, which were performed by an automatic tissue processor. Then the tissue sections (slices with 5-μm diameter) were prepared by a microtome, and for each kidney sample 10 slices were stained by hematoxylin and eosin staining protocol. The stained tissue samples were observed by an optical microscope (Olympus BX 51T 32E01 Optical, Tokyo, Japan) equipped with a DP12 camera (3.34 million pixel resolution) to examine the diameter and the number of glomeruli^[^^11^^]^.

**Griess** **technique**

Griess technique uses the zinc sulfate powder to remove the serum protein of the samples. Thus, zinc sulfate powder (6 mg) was mixed with serum samples (400 μl) and vortexed for 1 min. The samples were centrifuged at 7200 g at 4 °C for 10 min, and supernatant was used to measure the NO. In short, 50 μl of sample was added to 100 μl of Griess reagent (Sigma), and the reaction mixture was incubated at room temperature for about 30 min. According to the manufacturer protocol, an ELISA reader (Hyperion, USA) was applied to measure NO concentration at the wavelength of 450 nm^[^^22^^]^.

**Statistical analysis**

Kolmogorov–Smirnov test was performed to confirm the data compliance of the normal distribution after extracting the information. One-way analysis of variance (one-way ANOVA) was utilized for the statistical analysis of the data, and Tukey’s post hoc test was employed to determine the differences between the groups. SPSS software version 16 was applied to analyze the data, and the results were expressed as the mean ± standard error. *p* < 0.05 was considered statistically as significant.

## RESULTS

**Index of kidney**

**Fig. 1:**
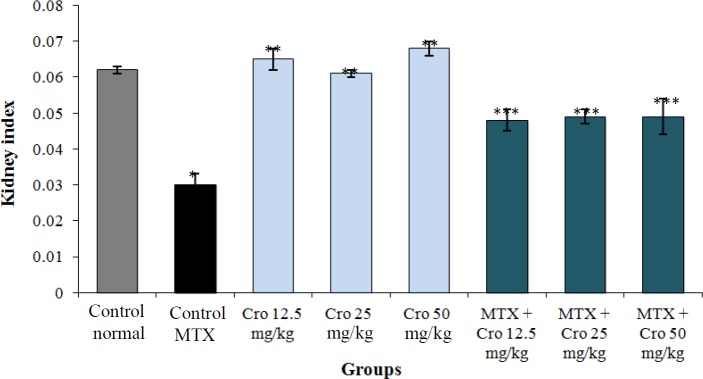
Effect of MTX, crocin (Cro), and MTX + Cro on renal index. ^*^Significant decrease in renal index of MTX group compared to the control group (*p* < 0.001); ^**^significant increase for all Cro groups compared to the MTX group (*p* < 0.001); ^***^Significant increase for all MTX + Cro groups compared to the MTX group (*p* < 0.001).

Crocin enhanced the renal index in all rat groups compared to the control MTX group (*p* < 0.001). The mean value of the index was insignificant in crocin groups in comparison to the control normal group (*p* > 0.05) and was improved significantly in crocin and crocin + MTX groups compared to the control MTX group (*p* < 0.001). Moreover, the MTX significantly reduced the mean value of the renal index relative to the normal control group (*p *< 0.001), as shown in Figure 1.

**Oxidative stress evaluation**

**Fig. 2:**
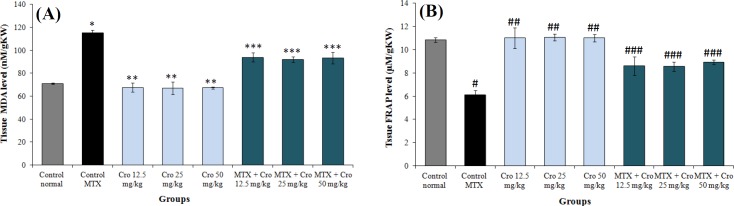
Comparing the MTX, control, and crocin (Cro) groups of (A) tissue MDA level and (B) tissue FRAP level. ^*^Significant increase in MTX control group compared to the control group (*p* < 0.001); ^**^significant decrease for all Cro groups compared to the MTX control group (*p* < 0.001); ^***^significant decrease for all MTX + Cro groups compared to the MTX group (*p* < 0.001); ^#^significant decrease in MTX group compared to the control group (*p* < 0.001); ^##^significant increase for all Cro groups compared to the MTX group (*p* < 0.001); ^###^significant increase for all MTX + Cro groups compared to the MTX group (*p* < 0.05).

The level of thiobarbituric acid reactive species significantly increased in the MTX groups relative to the control normal group (*p* < 0. 001) but reduced significantly in all crocin + MTX groups when compared to the MTX groups (*p* < 0.001). Similarly, the MTX significantly reduced the level of renal antioxidant capacity compared to the control normal group (*p* < 0.001). Administration of crocin significantly improved the level of the renal antioxidant capacity in all the crocin + MTX groups but not the MTX groups (*p* < 0.001). There was no significant crocin-related difference in the levels of antioxidant capacity of kidney in all groups in comparison to the control group (*p* > 0.05; Fig. 2). 

**Biochemical markers of renal function**

**Fig. 3:**
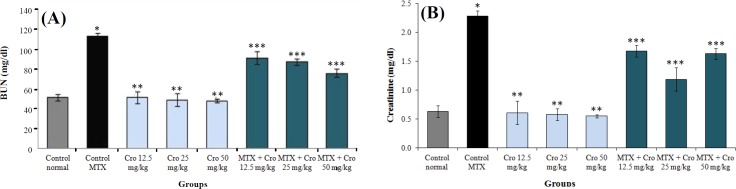
The effect of MTX, crocin (Cro), and MTX + Cro on the mean kidney biochemical factors. (A) BUN and (B) creatinine. ^*^Significant increase in biochemical factor in MTX control compared to normal control group (*p* < 0.001); ^**^Significant decrease in biochemical factor for all of crocin groups compared to MTX control group (*p* < 0.001); ^***^significant decrease in biochemical factors for all MTX + Cro groups compared to MTX group (*p* < 0.001)

MTX significantly enhanced the mean concentration of renal biochemical markers in comparison to the control group (*p* < 0.001). There was no significant concentration of renal biochemical markers in all crocin groups as compared to the control group (*p* > 0.05). However, a significant reduction was observed in the renal biochemical markers concentration in all crocin and crocin + MTX groups compared to the MTX control group (*p* < 0.001), as represented in Figure 3. 

**Morphometric and tissue examination**

**Fig. 4:**
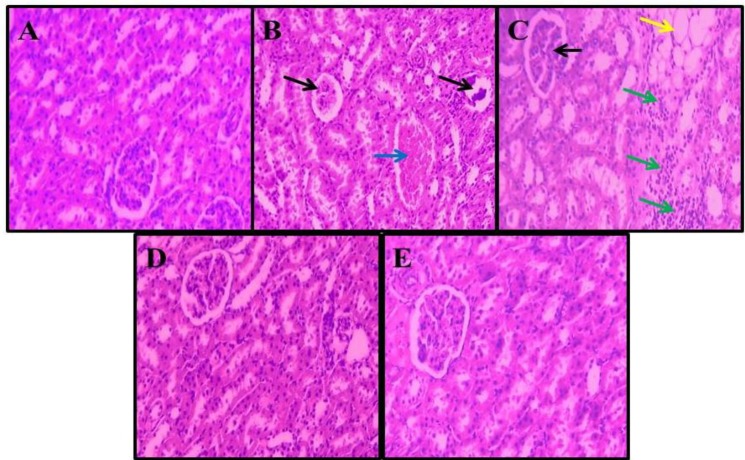
Histological changes in kidneys (hematoxylin-eosin, × 100). (A) Normal kidney, saline group; (B and C) MTX control group increased Bowman’s capsule space and glomerular shrinkage (black arrows) and distribution of lymphocytes (green arrows), bleeding in the space between the tubules (blue arrow), and formation of adipose tissue (yellow arrow); (D) normal kidney, crocin 50 mg/kg group; (E) normal kidney structure in MTX + crocin 50 mg/kg group

**Fig. 5:**
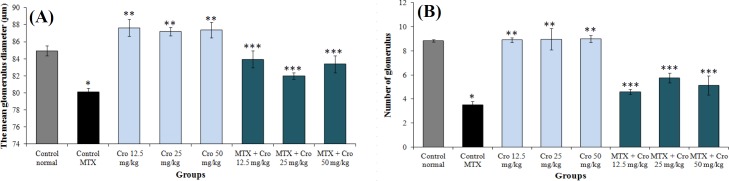
The relationship between treatment groups for (A) glomerular diameter and (B) glomeruli number. ^*^Significant increase in MTX group compared to the control group (*p* < 0.001); ^**^significant increase for all crocin groups compared to the MTX group (*p* < 0.001); ^***^significant increase for all MTX + crocin (Cro) groups compared to the MTX group (*p* < 0.001)

The normal renal structure was found in both the control and crocin groups. However, upon the MTX treatment, kidneys revealed obvious variations and injuries. A significant decrease was observed in several variables, including the number of Bowman's capsule, the amount of glomeruli as well as reduction in intertubular bleeding; besides, an increment was detected in proximal and distal tubules diameter. In all MTX + crocin groups, the renal damage reduced (Fig. 4). MTX significantly decreased the mean glomerulus and the number of glomeruli compared to the control normal group (*p* < 0.001). There was a significant enhancement in the glomeruli diameter treated with crocin (*p* < 0.001), and the changes in the number of glomeruli were not significant in all the treatment groups compared to the normal control group (*p* > 0.05). There was, however, a significant increase in the glomeruli diameter and glomeruli number in all crocin and MTX + crocin groups in comparison to the control MTX group (*p* < 0.001; Fig. 5).

**NO measurement**

**Fig. 6:**
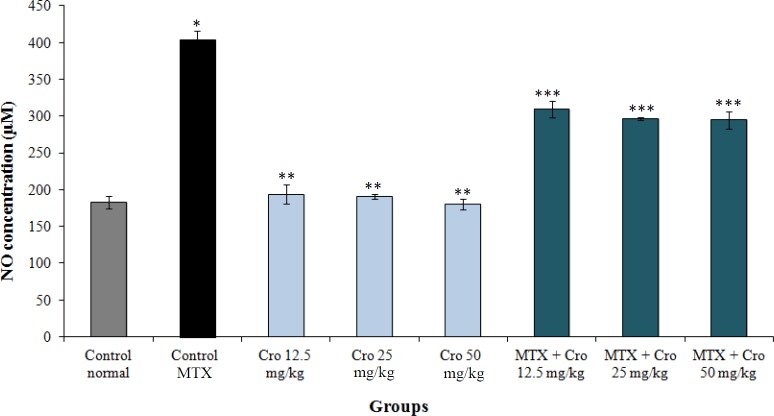
The effects of crocin, MTX, and MTX + crocin on the mean NO levels. ^*^Significant increase in NO in MTX group compared to the control group (*p* < 0.001); ^**^Significant decrease for all crocin groups compared to the MTX group (*p* < 0.001); ^***^Significant decrease for all MTX + crocin groups compared to the MTX group (*p* < 0.001)

The blood serum NO levels significantly enhanced in the MTX group compared to the control group (*p* < 0.001). There was no significant variation in the blood serum NO levels in all crocin groups in comparison to the normal control group (*p* > 0.001). However, a significant decrease was found in the mean of NO blood serum in all crocin and crocin + MTX groups as compared to the MTX group (*p* < 0.001; Fig. 6).

## DISCUSSION

Administration of MTX is a main approach for the treatment of some diseases; however, it accompanied with various side effects^[^^14^^]^. The MTX cytotoxicity and direct damage to the renal tubules are related to the generation of free radicals and oxidative stress^[^^13^^,^^23^^]^.

Our findings displayed a significant increase in the concentration of NO in all MTX groups in comparison to the control group. Besides, in all crocin + MTX groups, there was a significant reduction in NO serum levels compared to the MTX group. These results may confirm the antioxidant effects of crocin^[^^2^^]^. It is assumed that NO causes cell death by activating P53 pathway and cGMP and also by regulating the BCL2 and Bcl-xl expressions^[^^8^^]^. iNOS produces NO molecules in pathologic conditions, which can result in DNA production and degradation of many lipid and protein structures^[^^24^^]^. MTX has been suggested to cause cell damage and to induce apoptosis by the up-regulation of iNOS and production of NO^[^^25^^]^. The results of Leitão *et al.*'s^[^^26^^]^ study agreed with the results of this study, indicating that MTX could increase the iNOS expression. Crocin can inhibit the activity of NO by inducing the expression of HO-1 and calmodulin/calcium-dependent protein kinase-4^[^^27^^]^. Our findings was in line with Wang and Luo^[^^28^^]^ report, indicating that crocin can suppress the NO expression.

The outcomes of this study showed a significant reduction in glomeruli parameters of the MTX group in comparison to the control group but a significant increase in those of all MTX + crocin groups compared to the MTX group. Moreover, this study indicated the infiltration of lymphocytes by expanding Bowman's capsule space, diminishing the glomerular size, increasing the blood cells, and bleeding in the renal tubules in the MTX control group, which was reduced by crocin administration. Destructive effects of MTX include development of irregularity in glomerulus structure and damage to the proximal tubules. A decrease in the glomeruli parameters can be associated with the decrease in renal function^[^^29^^]^. In this study, by the use of crocin, we observed that there were only slight degenerative changes with no evidence of necrosis, which could show the protective effects of crocin extract against the MTX-induced toxicity. More than 90% of MTX is basically excreted through the kidneys^[^^30^^]^. It seems that renal toxicity is caused by the MTX precipitation, insoluble metabolites of MTX, or direct toxic effects of MTX on tubules^[^^31^^]^. According to the possible role of oxidative stress in MTX-induced damage, this substance likely accumulates the leukocyte in tissues by increasing the concentration of ROS. Active leukocytes secrete enzymes such as elastase, protease, and myeloperoxidase, which generate more free radicals^[^^32^^]^. ROS also increases the permeability in endothelial and epithelial cells^[^^33^^]; ^thus, it can damage cells due to MTX-induced oxidative stress by reacting with unsaturated fatty acids of cell membranes, DNA nucleotides, and sulfhydryl proteins bands. MTX can indirectly result in mitochondria damage, discharge of mitochondrial enzymes, and a reduction in antioxidant activity^[^^16^^]^. In addition, MTX activates Cdc42 and SAPK JNK by generating ROS and increases the expression of pre-apoptotic factors such as Bax^[^^34^^]^. The results of Asvadi *et al.*'s^[^^35^^]^ study were in line with the outcomes of our study, showing that the MTX significantly enhances the renal expression of TNF-α and alleviates the renal injury. Crocin can reduce the destructive effects of oxidative stress by inhibiting lipids and proteins peroxidation, preventing the decrease in glutathione levels, and increasing the antioxidant capacity, thus improving the cellular ability to cope with such demolition conditions^[^^3^^]^. Moreover, crocin seems to protect cells by regulating the inflammatory pathways and inhibiting the apoptosis^[^^11^^]^. 

 The results of a study conducted by Bie and Zheng^[^^36^^]^ have shown that crocin could inhibit the activity of apoptotic *Bcl2* gene in brain, which is in agreement with the results of this study. Our results also showed that crocin can reduce the lipid peroxidation (decreased MDA) and increase anti-oxidant capacity (increased FRAP) of renal tissue, thus reducing the oxidative stress levels.In parallel to these findings, several studies have shown the anti-oxidant properties of crocin^[^^2^^,^^3^^,^^8^^]^. Therefore, it appears that crocin could reduce the MDA and increase the FRAP in the treatment groups. The results of a study conducted by Asdaq^[^^37^^]^ matched with the outcomes of this study, indicating that crocin might decrease the MDA levels. 

Based on the results obtained from the present study, there was a significant increase in the markers of biochemical renal function in the MTX group compared to the control group. In all crocin and MTX + crocin groups, the BUN and creatinine reduced significantly compared to the MTX group. The results of a study conducted by Abdel-Daim *et al.*^[^^38^^]^ have also confirmed our results, which showed that MTX significantly increased the level of liver and kidney enzymes. MTX can produce the oxidative and nitrosative stress, thus causing the cellular damage by increasing the expression of NF-Kβ and P38 pathways^[^^17^^]^. Failed glomerular activity and renal parenchymal damage can enhance the BUN and creatinine levels^[^^20^^]^. Crocin stabilizes the cell membranes by preventing lipid peroxidation^[^^8^^]^. Similar to our results, Afolabi and Ugbaja^[^^39^^]^ observed that crocin significantly decreases liver enzymes. Our study showed that MTX-induced renal damage in rats could be reduced by herbal antioxidants such as crocin. In this sense, crocin could help individuals who have been exposed to MTX by protecting their kidneys. The antioxidant properties of crocin may be a main reason for its positive effect on kidney parameters; however, additional studies are required to define its exact mechanism of action.
